# Higher Pro-Inflammatory Dietary Score is Associated with Higher Hyperuricemia Risk: Results from the Case-Controlled Korean Genome and Epidemiology Study_Cardiovascular Disease Association Study

**DOI:** 10.3390/nu11081803

**Published:** 2019-08-05

**Authors:** Hye Sun Kim, Minji Kwon, Hyun Yi Lee, Nitin Shivappa, James R. Hébert, Cheongmin Sohn, Woori Na, Mi Kyung Kim

**Affiliations:** 1 Division of Cancer Epidemiology and Prevention, National Cancer Center, 323, Ilsan-ro, Ilsandong-gu, Goyang-si, Gyeonggi-do 10408, Korea; 2 Cancer Prevention and Control Program, University of South Carolina, Columbia, SC 29208, USA; 3 Department of Epidemiology and Biostatistics, Arnold School of Public Health, University of South Carolina, Columbia, SC 29208, USA; 4 Connecting Health Innovations LLC, Columbia, SC 29201, USA; 5 Department of Food and Nutrition, Wonkwang University, 460 Iksandaero, Iksan, Jeonbuk 54538, Korea

**Keywords:** hyperuricemia, dietary inflammatory index, inflammation, diet, public health

## Abstract

In previous studies, the elevated dietary inflammatory index (DII^®^) scores have been consistently associated with several chronic diseases. However, the relationship with hyperuricemia remains unknown. The aim of this study was to determine if the DII is associated with hyperuricemia risk. The study included 13,701 participants (men 5102; women 8599) in a large-scale cross-sectional study in South Korea. A validated semi-quantitative food frequency questionnaire (SQFFQ) was used to measure dietary intake, and blood samples were obtained to determine hyperuricemia. As the DII score increased, the hyperuricemia risk increased among women (OR 1.35, 95% CI 1.03–1.77, *p* trend = 0.02). However, no significant results were found for men. Women with lower BMI scores had higher risks of hyperuricemia with higher DII scores (OR 1.62, 95% CI 1.05–2.52, *p* trend = 0.03). As the DII increased, however, only women who consumed alcohol (“past or current drinkers”) had higher risks of hyperuricemia (OR 1.92, 1.22–3.02, *p* trend = 0.004). Among the DII components, intake of flavonoids showed a significant association with the hyperuricemia risk in women (OR 0.75, 0.59–0.96, *p* trend = 0.03). Our results suggest that higher intake of pro-inflammatory diet is significantly associated with higher risk of hyperuricemia among women. These results reinforce the importance of less pro-inflammatory habitual dietary patterns in lowering the risk of hyperuricemia and secondary afflictions such as cardiovascular diseases.

## 1. Introduction

Uric acid is the final product of purine metabolism in the human body. Hyperuricemia is the state of excessive uric acid retention. As hyperuricemia can be linked to major disease such as gout, chronic kidney disease and cardiovascular disease, strategies for its prevention are crucial for improved public health [1,2,3,4]. For example, it was found that 18.8% of hyperuricemic patients experienced gout within a follow-up period of five years [1], and that hyperuricemia was highly prevalent among those with renal failure [2]. Additionally, hyperuricemia was found to be strongly associated with cardiovascular disease [3].

Chronic inflammation causes a series of systemic reactions, including elevation of acute-phase proteins, that are related to cardiovascular and periodontal diseases [5,6,7]. As inflammation occurs, pro-inflammatory cytokines such as tumor necrosis factor-α (TNF-α), interleukin (IL)-1, and IL-6 are secreted from the inflammatory cells [5,6,7]. Uric acid activates the inflammatory NF-κB signaling pathway and induces inflammatory molecule expression [8,9].

Several studies have found that dietary habits influence the inflammation state [10,11,12,13]. The dietary inflammatory index (DII^®^), which measures a diet’s inflammatory potential, has been investigated to evaluate the validity of its association with inflammation markers. DII scores, which are calculated based on up to 45 food parameters, are based on literature examining the effect of dietary components on the level on six inflammatory markers (i.e., IL-1β, IL-6, C-reactive protein (CRP), IL-10, IL-4, and TNF-α), and was validated in studies with different inflammatory biomarkers (i.e., IL-6, high sensitivity CRP (hs-CRP), fibrinogen, homocysteine, and TNF-α) and populations [14,15,16,17,18,19,20,21,22,23,24]. Previous studies have found that individuals with high DII scores have significantly higher hs-CRP levels compared with those with low DII scores [14,15,16,17,18,19,20]. This demonstrates that the DII can be used to assess the association between diet’s inflammatory potential and chronic disease [21,22,23]. Because there have been no studies on the potential association of the DII with hyperuricemia risk, the present study used data obtained from the Korean Genome and Epidemiology Study (KoGES) to investigate this relationship. We hypothesized that subjects with higher DII scores, indicative of a more pro-inflammatory diet, will have a higher risk of hyperuricemia.

## 2. Materials and Methods

### 2.1. Study Population

Genetic, environmental, and lifestyle determinants of diseases (i.e., hyperuricemia, hypertension, obesity, osteoporosis, metabolic syndrome, cardiovascular disease, and cancer) among Koreans were obtained from KoGES, a general population-based prospective cohort study. Detailed information on its design and aims can be found elsewhere [25]. Among the various population-based cohorts in KoGES (e.g., KoGES_Ansan and Ansung Study, KoGES_Health Examinee [HEXA] Study, KoGES_Cardiovascular Disease Association Study [CAVAS]), KoGES_CAVAS data was used in the present study. The subjects’ were more than 40 years old at baseline, based on the National Health Examinee registry.

**Figure 1:**
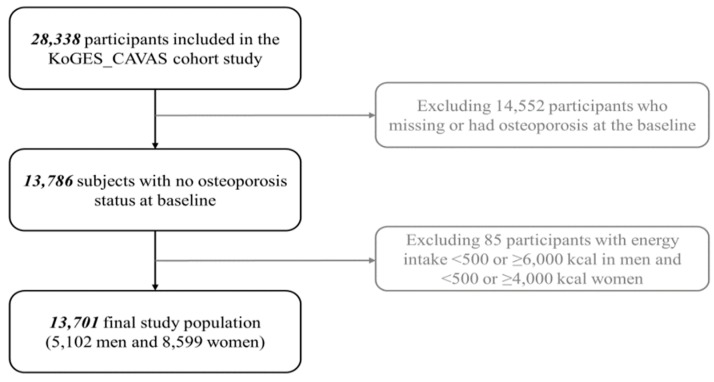
Flow chart of the study population. KoGES_CAVAS, Korean Genome and Epidemiology Study_Cardiovascular Disease Association Study.

Subjects were asked to volunteer for the study through media campaigns, conferences, letters, phone calls, and on-site invitations. A total of 13,701 participants (5102 men and 8599 women aged ≥40 years old) were recruited from 11 rural areas in Korea between 2005 and 2011. Of the original 28,338 willing participants, those who had an unreliable daily caloric intake (men <500 or >6000 kcal/day; women <500 or >4000 kcal/day) and/or no energy information were excluded, bringing the final sample size down to 13,701 (Figure 1). All of the study participants voluntarily signed a written informed consent before joining the study, and the study protocol was approved by the Institutional Review Board of the National Cancer Center (IRB No. NCC2018-0164) as well as the IRBs of the institutions that had participated in KoGES_CAVAS.

### 2.2. Data Collection

All of the study participants provided biomarkers according to standard procedures. The body height and weight of the participants were measured with subjects wearing light indoor clothing and without shoes. The body-mass index (BMI) was calculated as: weight (kg)/height (m)^2^. Blood pressure was measured on the right arm after resting in a quiet room for 5 min using a standard mercury sphygmomanometer (Baumanometer, W.A. Baum Co. Inc., Copiague, NY, USA). With each subject seated, a properly sized cuff placed along the mid-arm circumference at the heart level. The arm circumference was measured twice at 5 min intervals and the average of the two measurements was used.

Blood samples were collected from all of the study participants after at least 10 h of fasting. For long-term storage, the serum and plasma were separated and aliquoted in 6–10 vials (300–500 μL per vial), after which all of the samples were transported to the National Biobank of Korea [26]. Laboratory evaluations were performed in the same core clinical laboratory that is accredited and participates annually in inspections and surveys by the Korean Association of Quality Assurance for Clinical Laboratories. Blood concentrations of glucose, TC, HDL-cholesterol, and TG were measured using the enzyme method (ADVIA 1650 and ADVIA 1800; Siemens Healthineers, Deerfield, IL, USA). Serum concentrations of HDL-cholesterol and TG were determined using enzymatic methods (ADVIA 1650 Chemistry System, Bayer, Leverkusen, Germany). Hs-CRP was measured using a turbidimetric assay method (ADVIA 1650 and ADVIA 1800; Siemens Healthineers).

During the examination, sociodemographic information, family and personal medical history, nutrition intake, and physical activity consistency were obtained. Marital status was stratified into those who are married and those who are single/divorced/widowed/separated. Education status was divided into three categories: education up to elementary school, middle school to high school, and college education or above. Household income levels were divided into four categories: less than 1,000,000 won, between 1,000,000 and 2,000,000 won, between 2,000,000 and 3,000,000 won, and more than 4,000,000 won. With regard to smoking status, those who had smoked more than about 400 cigarettes and were continuing to smoke at the time of the survey were classified as “current” smokers; those who had smoked more than about 400 cigarettes but had stopped smoking by the time of the survey were designated as “past” smokers, and those who had not smoked more than 400 cigarettes were classified as “never” smokers. Drinking status was divided into three groups: Those who had consumed alcohol and were still drinkers at the time of the survey were deemed “current” drinkers; those who had consumed alcohol but were abstaining from drinking at the time of the survey were designated “past” drinkers, and those who had never consumed alcohol were classified as “never” drinkers. Regularity of physical activity was determined according to whether or not subjects participated regularly in any sports to the point of sweating. The history of diabetes and hypertension were indicated as “Yes” if diagnosed.

### 2.3. Diagnostic Criteria

Hyperuricemia was defined as a serum uric acid level of >7 mg/dL for men and >6 mg/dL for women, which are similar to the diagnostic criteria used in other studies, including in China [27,28,29]. A total of 1515 incident hyperuricemia cases were identified.

### 2.4. Dietary Assessment Using SQ-FFQ and Calculation of DII

To gather information on the subjects’ dietary intakes, they were asked to complete a validated semi-quantitative food frequency questionnaire (SQFFQ). Detailed information on the SQFFQ is available elsewhere [30,31]. Based on the SQFFQ, consumption frequencies and average amounts of 106 food items were determined. The frequencies were measured according to nine response categories ranging from “almost never” to “more than three per day.” Portion size was measured based on three responses including 1/2 serving, 1 serving, and 3/2 servings. Daily nutrient intake was estimated by combining obtained information on serving frequency per day, portion per unit for each food item, and average serving number. The food composition table of the Korean Health and Industry of Development was used in the calculation of nutrient intake and total energy [32].

Details on the DII are available elsewhere [33]. In short, a high DII score represents a pro-inflammatory diet and a low DII score represents an anti-inflammatory diet. For the updated DII, 1,943 research articles were reviewed and scored. In developing the DII, a total of 45 food parameters were scored based on their effects on the levels of six inflammatory markers (i.e., IL-1β, IL-6, CRP, IL-10, IL-4, and TNF-α). The DII has been validated in studies with different inflammatory biomarkers (i.e., interleukin (IL)-6, hs-CRP, fibrinogen, homocysteine, and TNF-α) and populations [14,15,16,17,18,19,20,21,22,23,24].

These pro-inflammatory parameters were included: total calories, cholesterol, carbohydrate, protein, total fat, saturated fatty acids, and vitamin B-12. These anti-inflammatory parameters were included: vitamin A, vitamin B-6, vitamin C, vitamin D, vitamin E, niacin, magnesium, riboflavin, beta-carotene, isoflavones, flavan-3-ol, flavanols, flavones, folic acid, fiber, alcohol, ginger, garlic, pepper, MUFAs, PUFAs, and tea. The present study utilized nutritional content data from the Functional Ingredients Table (Rural Development Administration), Computer Aided Nutritional Analysis (The Korean Nutrition Society), and the U.S. Department of Agriculture. From our SQFFQ 37 of the 45 parameters were available and these were as follows: carbohydrate, protein, fat, fiber, cholesterol, carotene, caffeine, energy, n-3 fatty acids, n-6 fatty acids, trans fat, saturated fat, mono-unsaturated fat, poly-unsaturated fat, niacin, thiamin, riboflavin, falvan-3-ol, flavones, flavonols, flavonones, anthocyanidins, isoflavones, vitamin B12, vitamin B6, iron, magnesium, zinc, selenium, vitamin A, vitamin C, vitamin D, vitamin E, folic acid, onion, garlic and tea. As a comparative standard for those parameters, a world database consisting of diet surveys from 11 countries was used [33]. The DII scores were calculated based on the database’s intake values, the details on which are available elsewhere [33]. In brief, a standard mean for each parameter from the database was subtracted from the actual individual exposure. The results were then divided by their standard deviation to generate Z scores. Those were converted to proportions to minimize the effects of outliers. The proportions were multiplied by two and then one was subtracted to achieve symmetrical distributions with values centered around 0. The results were then multiplied by the complementary inflammation score for each food parameter, which were all summed to obtain the overall DII score. In the DII score calculation, intakes from both foods and supplements were included. Several studies have found and validated the association between DII score and inflammatory markers in various populations [14,15,16,17,20,21,22,23,24].

### 2.5. Statistical Analysis

All of the statistical analyses were performed with SAS^®^ 9.3 (SAS Institute, Cary, NC, USA). For continuous variables such as age (years), energy intake (kcal), BMI, and hs-CRP levels, the results were expressed as medians (25%, 75%). Also, the following categorical variables were expressed as n (%). The chi-square test and *t*-test were used to compare categorical and continuous variables according to the case-control status of hyperuricemia. The DII score of the control group was divided into quartiles according to gender, and the range was applied to the group with hyperuricemia. The odds ratios and the corresponding 95% confidence intervals (ORs; 95% CIs) were estimated using logistic regression models, adjusting for age and additionally for smoking status, drinking status, education, BMI, daily caloric intake, hs-CRP, and region. Tests for trends were performed using DII as a continuous variable. Stratified analyses were carried out according to BMI and drinking status. The p value for interaction was calculated by contrasting the coefficients of the cross-product of BMI or drinking status and DII quartile in the multivariable logistic regression model. Flavonoid intake level of the DII component was divided into four levels in the control group in women. *p* values <0.05 were considered statistically significant.

## 3. Results

In the present study, 12,186 controls and 1515 hyperuricemia cases were included (Table 1). The DII scores were higher in female hyperuricemia cases than in controls. Compared with the controls, the cases were older, heavier (higher BMI), consumed fewer calories, had larger waist circumferences, and higher hs-CRP, homeostatic model assessment of insulin resistance (HOMA-IR), glucose and triglyceride. The percentages of past or current smokers and drinkers were higher in men with hyperuricemia than in women, while the hypertension-history rate was higher in women with hyperuricemia than in men.

The results of the multivariate logistic analysis suggested that people with hyperuricemia were more likely to be those in the highest quartile of the DII (Q4) (OR 1.23, 95% CI 1.03–1.46, *p* for trend = 0.02). The results also are reported by gender in Table 2. Among women, participants who had higher DII scores (Q4) had a significantly higher hyperuricemia risk (OR 1.35, 95% CI 1.03–1.77, *p* for trend = 0.02). No statistically significant association between DII and hyperuricemia risk was observed among the men (OR 1.10, 95% CI 0.87–1.39, *p* for trend = 0.49).

The association between hyperuricemia risk and DII score, as stratified by BMI and drinking status, was investigated (Table 3). Women with low BMI had higher risk of hyperuricemia for the DII quartiles (OR 1.62, 95% CI 1.05–2.52, *p* for trend = 0.03), while no association was found among those with high BMI scores (OR 1.19, 95% CI 0.84-1.68, *p* for trend = 0.34). However, a contrasting result was found among men, for whom there was no statistically significant association between hyperuricemia risk and DII score (OR 1.02, 95% CI 0.73–1.42, *p* for trend = 0.96; OR 1.18, 95% CI 0.85–1.67, *p* for trend = 0.27) in men with either a low or a high BMI score. Women drinkers showed a significant increase in hyperuricemia risk (OR 1.92, 95% CI 1.22–3.02, *p* for trend = 0.004), while no association was found among non-drinkers (OR 1.12, 95% CI 0.80–1.57, *p* for trend = 0.48). Among the men, both drinkers and non-drinkers showed only an insignificant increase in hyperuricemia risk according to their DII scores (OR 1.05, 95% CI 0.56–1.98, *p* for trend = 0.83; OR 1.12, 95% CI 0.86–1.42, *p* for trend = 0.49).

The association between flavonoids intake and hyperuricemia risk in women is shown in Table 4. High scores for flavan-3-ol, flavones, flavonols and flavonones were found to be significantly associated with decreased ORs for hyperuricemia risk in the women. Increased flavonoids, which represented the sum of those four components, were correlated with decreased risk of hyperuricemia (OR 0.75, 95% CI 0.59–0.96, *p* trend = 0.03).

## 4. Discussion

The present cross-sectional study based on data from the KoGES_CAVAS cohort aimed to explore the association between DII score, which represents diet-based inflammatory state, and hyperuricemia risk. The study found that higher DII scores (more pro-inflammatory diets) were associated with higher risk of hyperuricemia among women. Women in the highest DII score quartile (=Q4) had a 22% higher risk of hyperuricemia than those in the lowest DII score quartile (=Q1) after adjusting for potential confounders. The results of the current study were consistent with our hypothesis, which is that diet’s inflammatory potential can influence hyperuricemia risk. We assumed that in a state of inflammation, the body makes more uric acid or cannot excrete uric acid properly, which leads to hyperuricemia. In turn, hyperuricemia may worsen the state of inflammation, which condition can lead to gout or other autoimmune diseases. As the physiological mechanism of inflammation’s causative role with respect to hyperuricemia is not yet clearly known, future studies should be designed to address this issue.

The present study also found a statistically significant association between the DII and increased hyperuricemia risk among women with a BMI lower than 25 kg/m^2^; a finding which does not coincide with the results of previous studies that found a positive correlation between uric acid level and obesity [34,35,36,37]. However, other studies found that being either overweight or underweight was associated with higher inflammation in various populations, which might explain why women with low BMI scores had a significantly positive result for the relation of DII with hyperuricemia risk [38,39]. In any case, further research that can confirm the present results is called for.

Additionally, the present study found a statistically significant association between the DII and hyperuricemia risk among women drinkers. Previous studies had found that ethanol intake increases serum uric acid level via both decreased urate excretion and increased production [40,41,42,43]. Additionally, beer and serum uric acid are positively related due to beer’s high purine contents [42], which might explain the enhanced risk of hyperuricemia among those who consumed alcohol. Alcohol gets an anti-inflammatory score on the DII. Across all of the papers that were reviewed, alcohol, on average, exerted an anti-inflammatory effect. It is important to note that these effects are seen within the normal physiologic range. Of course, severe abuse of alcohol, which exerts a pro-inflammatory effect, is ill-advised. Additionally, when considering diet’s inflammatory potential, overall diet is key, and if people are consuming high amounts of alcohol, then it could mean that they are also consuming high amounts of carbohydrates and energy, which have pro-inflammatory scores on the DII.

The present study also found that hyperuricemia risk decreased as the quartile of flavonoids, which are DII components, increased. Flavonoids are products of the secondary metabolism of many edible plants. Flavonoids intake is inversely associated with cardiovascular disease, type 2 diabetes mellitus, hypertension, stoke risk, myocardial infarction, coronary heart disease, nonalcoholic fatty liver disease, dementia, and colorectal cancer [44,45,46,47,48,49,50,51,52,53]. Various studies on flavonoids suggest that they are antioxidants that are able to reduce free radical formation as well as the number of free radicals [54,55]. Oxidative stress is known to activate a variety of transcription factors, such as inflammatory cytokines, that can lead to the expression of various genes [56]. It is associated with activation of reactive oxygen species, which are supplemented by antioxidants such as flavonoids to reduce cellular oxidants, which, in turn, can prevent hyperuricemia. One study reported that flavonoids are related to ã-aminobutyric acid type A (GABA_A_) receptor for preventing alcohol use disorders [57]. We also performed a joint analysis of alcohol consumption and flavonoid intake for the risk of hyperuricemia. The results showed that the interaction of alcohol consumption and flavonoid intake was not significant (*p* = 0.23, data not shown), and the combined effect was significant, but it was similar to these factors’ respective single effects.

In the present study, the association between the DII and hyperuricemia risk differed by gender. While statistically significant results were shown among the women participants, no such results were observed among the men. Also in the subgroup analyses, statistically significant results were shown only among the women participants. Whereas there are no available studies on the association between the DII and the hyperuricemia risk that could be used for comparison, there has been some research stressing the importance of estrogen in promoting excretion of uric acid [58,59,60]. Estrogen, in fact, has been hypothesized to have a protective anti-inflammatory effect [61,62]. As the mean age of the present study’s women participants was older than the mean age at menopause among Korean women (49.2 years), the number of postmenopausal women in this study might have been the majority; if so, the results on hyperuricemia risk well might have been skewed higher [63]. Another important factor to consider is the role of tobacco smoke as a pro-inflammatory agent. It may be that the effect of tobacco swamps the effect of dietary sources of inflammation [64]. The fact that men smoked at about 10 times the rate of women in this study (75% of men were past or present smokers vs. only about 7% of women) may help to explain the sex differences observed in our study. More research on the issue of whether DII affects the risk of hyperuricemia only in women is needed.

Underlying our observations and understanding from all of the many studies that have been conducted to date using the DII are two countervailing effects [33,65,66]. The first is a tendency to eat more of everything as one increases caloric intake; this results in a positive correlation between caloric intake and nutrient intake. The other is the “healthy eater” effect (e.g., due to the intention of careful, health-conscious people to choose nutrient-dense, energy-sparse foods in preference to calorie-dense, nutrient-sparse foods). This type of eater produces data that results in negative correlations between caloric density and nutrient density. In this study, we anticipated the former to be happening; hence, we saw higher DII scores among women with hyperuricemia but lower values of not only pro-inflammatory components like energy and macronutrients but also fruits that contain vital micronutrients and other bio active compounds considered to be anti-inflammatory. In the DII calculation, there is no differentiation between complex and simple carbohydrates, and there is no overall inflammatory effect score for it, because when the literature search was done for carbohydrates, we did not find many articles demarcating them. A similar approach was used for proteins, as there was not enough research demarcating protein from animal proteins. However, these demarcations will again be considered in the next iteration of the DII, which will be developed sometime in the future.

There are three limitations to the present study, which should be duly considered in considering its results. First, the research participants were recruited in 11 rural areas of the National Health Examination Registry, but only those willing to participate were enrolled; consequently, more women enrolled than men; their proportion in this study does not represent the general population. This sort of screening bias occurs in many prospective cohort studies [67]. Second, possible confounding variables existed and could have affected the outcome. The present study adjusted for sex, smoking status, drinking status, education, BMI, age, daily caloric intake, CRP and region, which are potential confounding, as they affect both exposure and outcome. However, there may be variables that can affect hyperuricemia, such adiposity (expressed as a sum of skinfold thickness measurements) or creatinine levels [68]. Third, the questionnaire-derived data utilized in this study might not accurately reflect the subjects’ usual dietary intakes. The questionnaire contains a list of limited food items, and individuals cannot accurately report food intake retrospectively over a long period of time. Even with these limitations, the present study highlights the potential harmful effects of pro-inflammatory dietary patterns with respect to hyperuricemia risk. The results underline the importance of consuming a more anti-inflammatory diet as a strategy to lower the risk of hyperuricemia.

## Figures and Tables

**Table 1:** Characteristics of study participants in KoGES_CAVAS cohort.

	Male			Female		
Characteristics	With Hyperuricemia (*n* = 875)	Without Hyperuricemia (*n* = 4227)	*p* Value ^a^	With Hyperuricemia (*n* = 640)	Without Hyperuricemia (*n* = 7959)	*p* Value
Serum uric acid (mg/dL)	8.06 ± 0.99 ^b^	5.32 ± 1.00	<0.0001	6.90 ± 0.85	4.24 ± 0.87	<0.0001
DII	−0.01 ± 2.08	−0.08 ± 2.18	0.3610	0.46 ± 2.23	0.13 ± 2.16	0.0002
Age (years)	61.2 ± 10.1	62.2 ± 9.56	0.0035	64.2 ± 9.02	60.5 ± 9.94	<0.0001
BMI (kg/m^2^)	25.0 ± 3.10	23.8 ± 2.94	<0.0001	25.8 ± 3.64	24.4 ± 3.19	<0.0001
Waist circumference (cm)	88.4 ± 8.41	85.1 ± 8.37	<0.0001	86.5 ± 9.38	82.9 ± 8.93	<0.0001
hs-CRP (mg/L)	2.72 ± 6.48	2.19 ± 5.81	<0.0001	2.62 ± 4.48	1.58 ± 3.96	<0.0001
WBC (Thousand/ μL)	7.04 ± 1.94	6.76 ± 2.00	0.0001	6.99 ± 2.16	6.14 ± 1.79	<0.0001
Glucose (mg/dL)	102 ± 21.8	104 ± 28.8	0.0175	104 ± 24.3	98.2 ± 21.4	<0.0001
HOMA_IR	2.28 ± 2.05	1.86 ± 1.00	0.0265	2.53 ± 1.43	1.93 ± 0.96	<0.0001
SBP (mmHg)	127 (126–128)	125 (124–125)	0.0010	128 (127–130)	124 (123–124)	<0.0001
DBP (mmHg)	80.0 (79.3–80.8)	78.9 (78.6–79.2)	0.0027	78.5 (77.7–79.4)	76.8 (76.5–77.0)	<0.0001
Triglyceride (mg/dL)	199 (191–208)	154 (151–157)	<0.0001	193 (184–201)	141 (139–143)	<0.0001
HDL-cholesterol (mg/dL)	41.8 (41.0–42.5)	44.2 (43.8–44.5)	<0.0001	42.7 (42.0–43.5)	46.3 (46.0–46.5)	<0.0001
Daily caloric intake (kcal)	1694 (1661–1727)	1710 (1694–1726)	0.3980	1443 (1408–1477)	1494 (1484–1504)	0.0051
Carbohydrate intake (g/day)	308 (303–314)	315 (312–317)	0.0364	276 (269–282)	285 (284–287)	0.0030
Carbohydrate (E%)	73.7 (73.2–74.1)	74.4 (74.2–74.6)	0.0018	77.0 (76.5–77.5)	76.9 (76.8–77.0)	0.7963
Protein intake (g/day)	52.6 (51.2–54.0)	53.0 (52.1–53.5)	0.7806	42.7 (41.3–44.2)	44.4 (44.0–44.8)	0.1894
Protein (E%)	12.2 (12.1–12.4)	12.1 (12.1–12.2)	0.3754	11.7 (11.5–11.9)	11.8 (11.7–11.8)	0.7901
Fat intake (g/day)	24.8 (23.7–25.9)	23.8 (23.4–24.3)	0.1041	16.4 (15.4–17.3)	17.0 (16.7–17.3)	0.0237
Fat (E%)	12.4 (12.1–12.8)	11.9 (11.7–12.0)	0.0023	9.73 (9.35–10.1)	9.79 (9.68–9.89)	0.3891
Fruits (g/day)	146 (136–155)	152 (147–157)	0.2524	170 (157–183)	184 (180–188)	0.0386
Vegetables (g/day)	213 (203–222)	235 (230–240)	<0.0001	184 (172–195)	207 (304–210)	0.0001
Marriage						
Married	811 (93.2)	3987 (94.8)	0.07	422 (66.0)	5932 (74.8)	<0.0001
Single	59 (6.8)	220 (5.2)		217 (34.0)	1999 (25.2)	
Education						
~Elementary school	397 (45.5)	1998 (47.4)	0.19	474 (74.3)	5524 (69.6)	0.03
Middle~High school	379 (43.5)	1831 (43.5)		142 (22.2)	2149 (27.1)	
College~	96 (11.0)	383 (9.1)		22 (3.5)	270 (3.3)	
Household Income Levels ^c^						
Less than 100	72 (38.1)	366 (38.1)	0.58	97 (57.7)	885 (46.9)	0.01
100~less than 200	38 (20.1)	234 (24.4)		32 (19.1)	383 (20.3)	
200~less than 300	35 (18.5)	160 (16.7)		22 (13.1)	263 (13.9)	
More than 300	44 (23.3)	200 (20.8)		17 (10.1)	356 (18.9)	
Smoking Status						
Never	217 (24.8)	1070 (25.3)	0.07	575 (89.8)	7578 (95.3)	<0.0001
Past	397 (45.4)	1751 (41.4)		25 (3.9)	144 (1.8)	
Current	261 (29.8)	1404 (33.3)		40 (6.3)	234 (2.9)	
Drinking Status						
Never	135 (15.5)	1011 (23.9)	<0.0001	416 (65.10)	5404 (68.0)	0.0008
Past	114 (13.0)	606 (14.4)		41 (6.42)	278 (3.5)	
Current	626 (71.5)	2607 (61.7)		182 (28.48)	2268 (28.5)	
Physical Activity ^d^						
No	594 (67.9)	2842 (67.2)	0.72	435 (68.0)	5526 (69.5)	0.43
Yes	281 (32.1)	1384 (32.8)		205 (32.0)	2429 (30.5)	
History of Hypertension						
No	556 (63.5)	3193 (75.6)	<0.0001	297 (46.4)	5749 (72.2)	<0.0001
Yes	319 (36.5)	1033 (24.4)		343 (53.6)	2209 (27.8)	
History of Diabetes						
No	787 (89.9)	3769 (89.2)	0.51	551 (86.1)	7270 (91.4)	<0.0001
Yes	88 (10.1)	457 (10.8)		89 (13.9)	688 (8.6)	

DII, Dietary inflammatory index; BMI, body-mass index; hs-CRP, high-sensitivity C-reactive protein; WBC, white blood cell; SBP, systolic blood pressure; DBP, diastolic blood pressure; HOMA-IR, homeostasis model assessment insulin resistance (fasting insulin × fasting plasma glucose/405); HDL-cholesterol, high-density lipoprotein cholesterol. ^a^ Comparisons between groups without and with hyperuricemia were performed by *t*-test or Chi-squared tests. ^b^ The data are presented as mean ± standard deviation (SD) or mean (95% confidence interval) for continuous variables and as n (%) for categorical variables. ^c^ 10,000 Korean won; 1000 won is about 850 dollars or about the same value in euros. ^d^ Regularity of physical activity was determined according to whether or not subjects participated regularly in any sports to the point of sweating.

**Table 2:** Odds ratios (ORs) for hyperuricemia and corresponding 95% CI by quartile of DII scores in KoGES_CAVAS cohort.

	Quartile of DII					
	First (Lowest)	Second	Third	Fourth (Highest)	*p* for Trend ^a^	Continuous DII
**TOTAL**						
Range	−7.3344~−1.2409	−1.2408~−0.2501	−0.2499~1.5499	1.5513~7.0740		
Cases/controls	349/3047	367/3047	391/3047	408/3045		
Odds ratio (95% CI)						
Crude	1.00	1.05 (0.90−1.23) ^b^	1.12 (0.96–1.31)	1.17 (1.01–1.36)	0.03	1.03 (1.00–1.05)
Multivariate adjusted	1.00	1.05 (0.89–1.25)	1.01 (0.92–1.30)	1.23 (1.03–1.46)	0.02	1.04 (1.01–1.07)
**MEN** ^c^						
Range	−7.3344~−1.3572	−1.3565~−0.3522	−0.3520~1.4001	1.4012~6.9009		
Cases/controls	212/1057	217/1057	220/1057	226/1056		
Odds ratio (95% CI)						
Crude	1.00	1.02 (0.83–1.26)	1.04 (0.84–1.28)	1.07 (0.87–1.31)	0.53	1.02 (0.98–1.05)
Multivariate adjusted	1.00	1.03 (0.82–1.29)	1.00 (0.80–1.26)	1.10 (0.87–1.39)	0.49	1.02 (0.98–1.06)
**WOMEN**						
Range	−7.3009~−1.1924	−1.1915~−0.1928	−0.1922~1.6153	1.6154~7.0740		
Cases/controls	126/1990	148/1989	170/1991	196/1989		
Odds ratio (95% CI)						
Crude	1.00	1.18 (0.92–1.50)	1.35 (1.06–1.71)	1.56 (1.23–1.96)	<0.0001	1.07 (1.03–1.11)
Multivariate adjusted	1.00	1.11 (0.85–1.45)	1.17 (0.90–1.52)	1.35 (1.03–1.77)	0.02	1.04 (0.99–1.01)

Q1 indicates participants having the lowest DII values, the least pro-inflammatory level; Q4 the highest, the most pro-inflammatory level. DII, Dietary inflammatory index. ^a^ Tests for trends were performed for continuous variable using categorical DII scores by quartiles. ^b^ Data are presented as odds ratios (ORs) with correspondent 95% confidence intervals (CI) and are adjusted for gender (for total subjects), smoking status, drinking status, education, body-mass index (BMI), age, caloric per day, history of diabetes, history of hypertension, and region. ^c^ p value for interaction was calculated as the cross-product of gender (men and women) and the continuous DII score in the model was 0.20.

**Table 3:** Multivariable adjusted ORs of hyperuricemia and corresponding 95% CI by quartile of DII scores stratified by BMI, drinking in KoGES_CAVAS cohort.

	Quartile of DII				*p* for		
Subgroup	First (Lowest)	Second	Third	Fourth (Highest)	Trend ^a^	Interaction ^b^	Continuous DII
**TOTAL**							
Body-mass index							
Cases/controls	134/1747	182/1896	182/1906	216/1974			
<25	1.00	1.11 (0.86–1.42) ^c^	1.04 (0.81–1.34)	1.25 (0.97–1.62)	0.12	0.56	1.04 (0.99–1.09)
Cases/controls	214/1299	185/1150	209/1141	192/1069			
>=25	1.00	1.02 (0.81–1.29)	1.15 (0.91–1.44)	1.20 (0.94–1.54)	0.09		1.03 (0.99–1.07)
Drinking status							
Cases/controls	105/1460	141/1626	155/1610	150/1719			
No	1.00	1.13 (0.85–1.50)	1.23 (0.93–1.63)	1.13 (0.84–1.52)	0.41	0.24	1.02 (0.97–1.07)
Cases/controls	244/1586	226/1415	235/1436	258/1322			
Yes ^d^	1.00	1.03 (0.83–1.27)	1.03 (0.83–1.27)	1.32 (1.06–1.63)	0.02		1.05 (1.01–1.09)
**MEN**							
Body–mass index							
Cases/controls	89/602	115/708	109/722	130/751			
<25	1.00	0.98 (0.72–1.35)	0.86 (0.62–1.19)	1.02 (0.73–1.42)	0.96	0.70	1.01 (0.96–1.07)
Cases/controls	122/455	102/348	111/335	96/305			
>=25	1.00	1.10 (0.79–1.52)	1.17 (0.85–1.61)	1.18 (0.85–1.67)	0.27		1.03 (0.97–1.09)
Drinking status							
Cases/controls	26/246	37/252	44/249	28/264			
No	1.00	1.42 (0.79–2.53)	1.73 (0.98–3.07)	1.05 (0.56–1.98)	0.83	0.08	1.01 (0.92–1.12)
Cases/controls	186/810	180/803	176/808	198/792			
Yes	1.00	0.97 (0.76–1.25)	0.91 (0.71–1.17)	1.12 (0.86–1.42)	0.49		1.02 (0.98–1.07)
**WOMEN**							
Body-mass index							
Cases/controls	41/1146	61/1173	74/1192	95/1229			
<25	1.00	1.26 (0.81–1.95)	1.33 (0.87–2.06)	1.62 (1.05–2.52)	0.03	0.67	1.09 (1.01–1.16)
Cases/controls	85/843	87/816	96/799	101/758			
>=25	1.00	1.02 (0.73–1.43)	1.09 (0.78–1.52)	1.19 (0.84–1.68)	0.34		1.01 (0.96–1.07)
Drinking status							
Cases/controls	82/1265	101/1362	113/1359	120/1418			
No	1.00	1.06 (0.76–1.48)	1.11 (0.80–1.53)	1.12 (0.80–1.57)	0.48	0.21	1.01 (0.96–1.07)
Cases/controls	44/725	47/623	56/631	76/567			
Yes	1.00	1.22 (0.77–1.94)	1.31 (0.84–2.06)	1.92 (1.22–3.02)	0.004		1.11 (1.03–1.19)

Q1 indicates participants having the lowest DII values, the least pro-inflammatory level; Q4 the highest, the most pro-inflammatory level. DII, Dietary inflammatory index. ^a^ Tests for trends were performed for continuous variables using categorical DII scores by quartiles. ^b^ P for interaction was calculated by contrasting the coefficients of the cross-product of stratified values and DII quintiles in the model. ^c^ Data are presented as odds ratios (ORs) with correspondent 95% confidence intervals (CI) and are adjusted for gender (for total subjects), smoking status, drinking status, education, body-mass index (BMI), age, caloric per day, history of diabetes, history of hypertension, and region. ^d^ Represents past or current drinking.

**Table 4:** Multivariable adjusted ORs of hyperuricemia and corresponding 95% CI by quartile of flavonoids of DII components in women in KoGES_CAVAS cohort.

	Quartile of Components				
DII components (Flavonoids)	First (lowest)	Second	Third	Fourth (highest)	P for trend ^a^
Flavan-3-ol (mg)					
Cases/controls	190/1990	167/1990	147/1990	136/1989	
Multivariate adjusted	1.00	0.89 (0.71–1.12) ^b^	0.85 (0.68–1.08)	0.78 (0.61–0.99)	0.04
Flavones (mg)					
Cases/controls	183/1990	163/1989	167/1990	127/1990	
Multivariate adjusted	1.00	0.95 (0.76–1.20)	0.97 (0.77–1.22)	0.75 (0.59–0.96)	0.04
Flavonols (mg)					
Cases/controls	181/1989	164/1990	166/1990	129/1990	
Multivariate adjusted	1.00	0.94 (0.75–1.18)	0.98 (0.78–1.23)	0.76 (0.59–0.97)	0.06
Flavonones (mg)					
Cases/controls	196/1989	150/1991	161/1989	133/1990	
Multivariate adjusted	1.00	0.83 (0.66–1.05)	0.89 (0.71–1.11)	0.75 (0.59–0.95)	0.04
Flavonoids (mg)					
Cases/controls	187/1990	165/1989	156/1991	132/1989	
Multivariate adjusted	1.00	0.92 (0.74–1.16)	0.90 (0.71–1.13)	0.75 (0.59–0.96)	0.03

Q1 indicates participants having the lowest DII values, the least pro-inflammatory level; Q4 the highest, the most pro-inflammatory level. DII, Dietary inflammatory index; Flavonoids = flavan-3-ol+flavones+flayonols+flayonones. ^a^ Tests for trends were performed for continuous variables using categorical component scores by quartiles. ^b^ Data are presented as odds ratios (ORs) with correspondent 95% confidence intervals (CI) and are adjusted for smoking status, drinking status, education, body-mass index (BMI), age, history of diabetes, history of hypertension, and region.
